# A17 COMPARING ENDOSCOPIST DIAGNOSIS OF COLORECTAL POLYPS ASSISTED BY ARTIFICIAL INTELLIGENCE (CADX) VS CADX WITHOUT ENDOSCOPIST INPUT: A RANDOMIZED CONTROLLED TRIAL

**DOI:** 10.1093/jcag/gwad061.017

**Published:** 2024-02-14

**Authors:** R Djinbachian, C Haumesser, M Taghiakbari, A Alj, A Barkun, J Liu, B Panzini, S Sidani, D von Renteln

**Affiliations:** CRCHUM, Montréal, QC, Canada; CRCHUM, Montréal, QC, Canada; CRCHUM, Montréal, QC, Canada; CRCHUM, Montréal, QC, Canada; McGill University, Montreal, QC, Canada; CRCHUM, Montréal, QC, Canada; CRCHUM, Montréal, QC, Canada; CRCHUM, Montréal, QC, Canada; CRCHUM, Montréal, QC, Canada

## Abstract

**Background:**

Artificial intelligence-based optical diagnosis systems (CADx) have been developped to assist in eliminating the need for histologic diagnosis of diminutive colorectal polyps (resect-and-discard and diagnose-and-leave strategies). However, these systems have not yet been implemented in routine clinical practice.

**Aims:**

We were interested in evaluating the performance of CADx without human input to diagnoses performed by endoscopists assisted by CADx.

**Methods:**

We performed a randomized clinical trial of patients undergoing elective colonoscopies at the CHUM. Patients were randomized into two arms: 1) optical diagnosis of colorectal polyps using CADx without human input; 2) endoscopists performed optical diagnosis after consulting a real-time CADx diagnosis (Human in the Loop [HiL]). Primary outcome was accuracy in optical diagnosis for both arms.

**Results:**

467 patients were randomized (229 in the CADx group, 238 in the HiL group). Overall accuracy for optical diagnosis was 76.3% in the CADx group and 70.5% in the HiL group (p=0.19). Sensitivity, specificity, PPV and NPV for adenoma diagnosis were 89.7%, 58.4%, 82.2%, and 72.5% respectively in the CADx group vs 85.3%, 69.6%, 81.8%, and 74.8% in the HiL group. Sensitivity, specificity, PPV and NPV did not differ significantly between the two groups.

**Conclusions:**

Optical diagnosis of colorectal polyps had similar accuracy when using CADx without human input compared to endoscopist-based diagnosis assisted by CADx. Resect and discard and diagnose and leave strategies can therefore potentially be implemented without need for endoscopist optical diagnosis.

**Funding Agencies:**

CAGFujifilm

